# Advocacy counterstrategies to tobacco industry interference in policymaking: a scoping review of peer-reviewed literature

**DOI:** 10.1186/s12992-023-00936-7

**Published:** 2023-06-21

**Authors:** Britta K. Matthes, Praveen Kumar, Sarah Dance, Tom Hird, Angela Carriedo Lutzenkirchen, Anna B. Gilmore

**Affiliations:** 1 Department for Health, University of Bath, Claverton Down, Bath, BA27AY UK; 2 Manipal Academy of Higher Education, Manipal, India

**Keywords:** Tobacco industry interference, Civil society, Advocacy, Tobacco control

## Abstract

**Background:**

There has been remarkable tobacco control progress in many places around the globe. Tobacco industry interference (TII) has been identified as the most significant barrier to further implementation of the World Health Organization Framework Convention on Tobacco Control (WHO FCTC). Civil society has been recognised as a key actor in countering TII. While TII has been extensively studied for several decades now, there is little research that focuses on counteractions to limit it and their effectiveness to do so. This scoping review seeks to map the peer-reviewed literature on civil society’s activities of countering TII in policymaking to identify common counterstrategies and assess their effectiveness.

**Methods:**

Data sources: We searched Embase, IBSS, JSTOR, PubMed, Science Direct, Scopus and Web of Science using the following terms: (“Tobacco industry” OR “Tobacco compan*”) AND. (“corporate political activity” OR “CPA” OR “lobbying” OR “interference”) AND (“advoca*” OR “counter*” OR “activi*”), without time or language restrictions. Study selection: Our selection criteria included peer-reviewed studies that were written in English, German, or Spanish that drew on primary data and/or legal and policy documents and reported at least one specific example of civil society members or organisations countering tobacco industry action-based strategies. Data extraction: Advocates’ counterstrategies were analysed inductively and countered industry strategies were analysed using the Policy Dystopia Model (PDM). Perceptions of effectiveness of countering attempts were analysed descriptively.

**Results:**

We found five common counterstrategies among 30 included papers covering five WHO regions; 1. Exposing industry conduct and false claims; 2. Accessing decision-makers; 3. Generating and using evidence; 4. Filing a complaint or taking legal action; 5. Mobilising coalition and potential supporters. These counterstrategies were used to work against a wide range of industry strategies, which are captured by five action-based strategies described in the PDM (Coalition Management, Information Management, Direct Access and Influence, Litigation, Reputation Management). While some studies reported the outcome of the countering activities, their impact remained largely underexplored.

**Conclusion:**

The review shows that peer-reviewed literature documenting how civil society actors counter TII is scarce. It suggests that advocates employ a range of strategies to counter TII in its different forms and use them flexibly. More work is needed to better understand the effects of their actions. This could stimulate discussions about, and facilitate learning from, past experiences and help to further enhance advocates’ capacity.

**Supplementary Information:**

The online version contains supplementary material available at 10.1186/s12992-023-00936-7.

## Background

Civil society is a crucial actor in policymaking, operating as advocate, provider of evidence-based information, coalition builder and watchdog [1]. Tobacco control is no exception: members of civil society raise awareness of the harms of smoking in the general population, generate and disseminate evidence, and form alliances and operate in coalitions. They also seek to pressure policymakers to act in the public interest and expose industry conduct, including violations of existing regulations and attempts to influence future policies. The World Health Organization Framework Convention on Tobacco Control (WHO FCTC) recognises in its Guiding Principles that advocacy is vital for achieving the treaty’s objectives [2].

While there has been remarkable progress in tobacco control policies in many places around the globe [3], tobacco industry interference (TII) remains a key obstacle to tobacco control policymaking and is identified as the most significant barrier to further implementation of the WHO FCTC [4–6]. Civil society plays an important role in countering and overcoming TII: In the grey literature, examples of advocates successfully countering TII can be found [7, 8] and case studies describe how advocacy contributed to policy progress which required overcoming TII [9–11]. Furthermore, an abundance of material has been developed that describe common industry tactics and offers advice on how to identify, monitor, expose, and prevent TII [12–15]. Resources also list common industry arguments and how these can be and have been countered [16–21].

The vast majority of peer-reviewed literature on TII has so far remained focused on industry strategies to block or undermine policies that threaten its profits. These include lobbying policymakers, fabricating industry-favourable evidence, astroturfing, and taking legal action [22–25]. The accumulation of hundreds of case studies on TII from across the world has enabled a detailed understanding of these industry tactics. In contrast, a small number of peer-reviewed case studies on tobacco control policies are framed around advocates’ work, although the number is growing [26–30]. Given the significance civil society actions have had in advancing the tobacco control agenda [11, 31], exploring these and other relevant studies in more depth is needed in order to further understand how TII is countered. This could support knowledge sharing [32] and it also allows us to critically reflect on the state of the literature and identify areas requiring more attention.

With the present review, we seek to map the existing peer-reviewed literature on civil society’s actions against TII before and after policy adoption. We focus on the countering of action-based strategies which has to date received less attention than the countering of argument-based strategies [16–21, 33, 34]. We aim to explore 1) how advocates counter TII in the context of public health policymaking, 2) which tobacco industry action-based strategies advocates counter with what counterstrategies, and 3) to what extent the advocates’ countering attempts are reported as being effective.

## Methods

This is an exploratory study that seeks to map and summarise the literature on civil society countering of TII in policymaking. As a scoping review, this study aims to synthesise the existing evidence on the topic and highlight gaps in the body of literature [35–37].

We used the Preferred Reporting Items for Systematic Reviews and Meta-Analyses’ Extension for Scoping Reviews (PRIMA-ScR) [38] when preparing, conducting, and writing up this review (checklist in online supplement).

### Study identification and selection

#### Search strategy

We used the following search terms: (“Tobacco industry” OR “Tobacco compan*”) AND (“corporate political activity” OR “CPA” OR “lobbying” OR “interference”) AND (“advoca*” OR “counter*” OR “activi*”). These search terms were included on the basis of key terms of the project and linked terms. Boolean operators were added to combine terms.

We searched seven databases: Embase, International Bibliography of the Social Sciences (IBSS), JSTOR, PubMed, ScienceDirect, Scopus, and Web of Science. We included databases focused on health literature (e.g., PubMed) as well as databases (e.g., IBSS) that capture social science research to ensure we identified literature within and beyond public health.

Searches were conducted in April 2021 without time or language restrictions. Two researchers (PK and BM) searched independently, compared their results, and resolved any discrepancies.

#### Eligibility criteria

We excluded studies that were a) not written in English, German, or Spanish (due to the researchers’ language proficiency), b) unrelated to or not relevant to TII which included fields such as chemistry, pharmacology, and bioelectronics, and c) books, conference abstracts, literature reviews, letters, Industry Watch pieces, and interviews.

To be included, a study had to a) be peer-reviewed, drawing primarily on primary data and/or legal and policy documents, and b) report at least one specific example of one or more civil society member or organisation countering tobacco industry action-based strategies aimed at avoiding, pre-empting, weakening, delaying or undermining a tobacco control policy.

#### Study selection

All references that appeared as search results were imported into EndNote X.9 3.3. and duplicates were removed. Next, titles and, if available, abstracts of the remaining references were screened by two researchers (PK and BM) to verify they were related to tobacco. The full texts of the remaining studies were sought. Then, both researchers screened the full texts to exclude those that met one or more of the exclusion criteria.

To ensure that coders developed a common understanding of the inclusion criteria’s application, we ran two rounds of pilots. In each round, three researchers (BM, PK and SD) first independently assessed six studies and then compared and discussed their assessments. BM selected the papers for the pilots to ensure that some were potentially included so that discussions were relevant. Next, BM, PK and SD assessed if the remaining studies met the inclusion criteria. For this, the full texts were read, and each study was independently assessed by two researchers. Intercoder-reliability was 81.8% which was calculated based on an Excel v2102 sheet in which the lead researcher (BM) recorded the entire coding process. Discrepancies were discussed and resolved in meetings of all three coders and decisions were added to the Excel sheet.

The emphasis on primary data meant that we did not include studies that referred to examples presented by others [39] or those that offered recommendations on how advocates should counter TII [40–43]. Concentrating on civil society members and organisations meant papers documenting how policymakers or public officials countered TII [44–46] were ineligible. Finally, as we emphasised TII in policymaking, we did not include studies documenting activities around public tobacco control programmes [47–49], counter-marketing activities [50–52], pre-emptive or preventive action [53–56] or studies documenting tobacco control activities more generally [57, 58].

#### Additional searches

Additional searches, carried out in June 2021, included reviewing the reference lists of the included articles and conducting forward-searches using Google Scholar’s cited-by function. The latter was repeated in March 2023.We also conducted follow-up searches using Google Scholar in March 2023.

### Data charting and analysis

Data charting, facilitated by Excel v2102, consisted of three steps. First, we focused on advocates’ strategies to counter TII. The categorisation of counterstrategies was developed inductively. Three researchers (BM, PK and SD) read all relevant extracts and developed independently lists of categories. These lists were discussed in a meeting which resulted in an agreed draft categorisation which was refined in subsequent meetings.

**Fig. 1:**
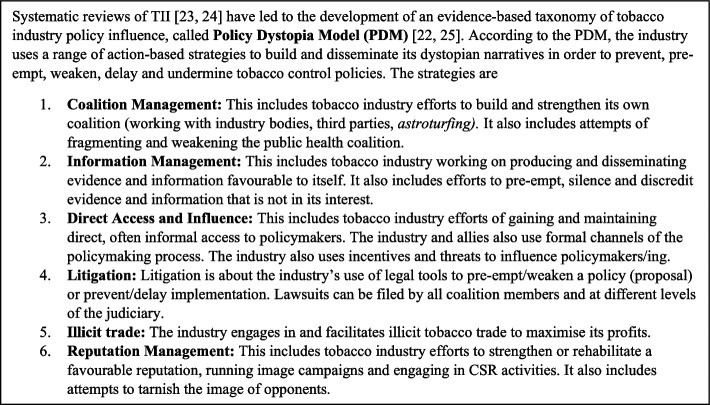
The Policy Dystopia Model and its action-based strategies

Second, for the industry strategies advocates countered, we used the action-based industry strategies described in the Policy Dystopia Model (PDM) (see Fig. 1) [22, 25] as a starting point. Additional strategies that did not feature in the PDM could be added.

Three researchers (BM, PK and SD) coded all extracts independently to industry strategies and counterstrategies and discussed all coding in meetings until consensus was reach. If more than one strategy was mentioned, the extract was coded to all relevant strategies. Then, we mapped counterstrategies against industry strategies.

Finally, considering the effectiveness of the countering TII, we looked at two standard criteria used in programme and policy evaluations: outcomes and impact [59]. For this study, outcomes were the immediate effects of the countering activities on TII (e.g., invitation to a meeting with industry declined, an industry-friendly statement withdrawn, a donation returned) that had the potential to contribute to long-term changes (e.g., improved tobacco control legislation). Impacts were the observed long-term effects of the countering activities on TII or their outcomes in a given context. To analyse outcomes and impacts of countering activities, we revised the extracts and additional information from the papers, and categorised them accordingly.

## Results

**Fig. 2:**
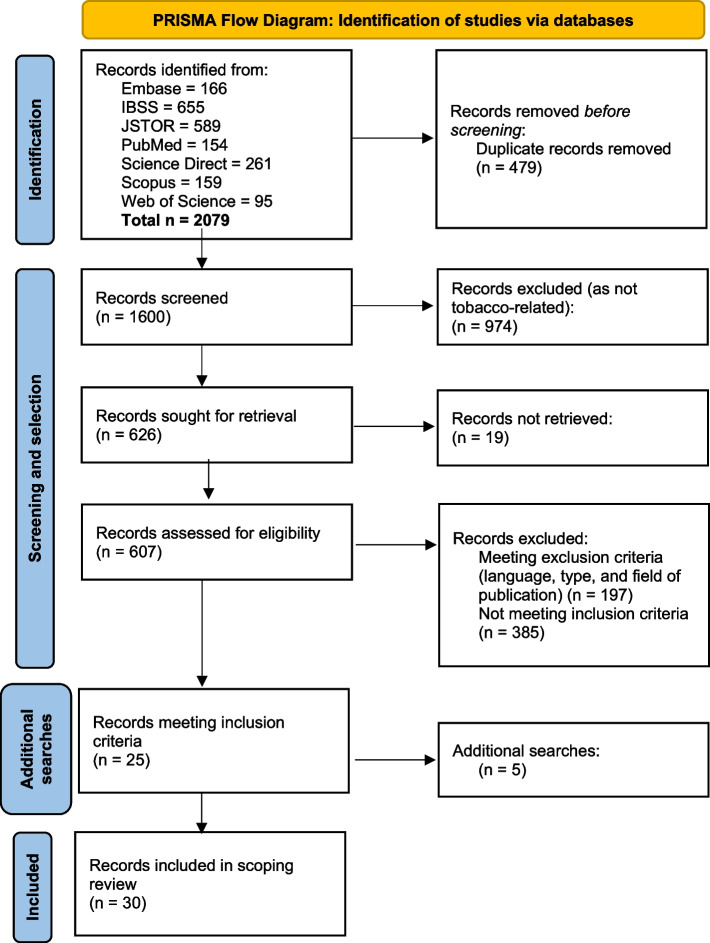
Prisma flow diagram (based on Page et al. 2021 [60])

Figure 2 presents the PRISMA flow diagram [60] of this review (see Additional file 1 for a list of included studies).

All 30 studies were published between 1997 and 2021: three in the late 1990s, eight between 2000 and 2010, and the remaining 19 after 2010, including 15 after 2015.

Countering activities described in 13 papers related to high-income countries (HICs) whereas 17 were related to low- and middle-income countries (LMICs). Out of the 13 HIC-focused studies, ten reported instances of countering TII in the USA. Of the LMIC-focused papers, three were on Thailand [26, 61, 62] and Nepal [27, 63, 64], and two were on Costa Rica [65, 66] and Mexico [67, 68] each. Apart from three articles [69–71], the HIC-based studies were published between 1997 and 2010 and of the LMIC-based studies all bar one [61] were published after 2010.

Considering the geographical spread, 17 studies described countering activities focused on countries in the Americas region, including 11 on the USA and Canada, six were located in South-East Asia, three in Africa, two in Europe and one each in the Eastern Mediterranean and Western Pacific regions. One study [32] covered eight countries in four WHO regions but did not specify where which instance took place.

## How do advocates counter industry interference?

Advocates repeatedly used five counterstrategies in response to TII in policymaking, with an additional strategy mentioned in one study (see Table 1). The findings for each counterstrategy are summarised below.

**Table 1:** Counterstrategies of advocates

**Countering strategy**	**No of papers**^**a**^	**Papers mentioning example(s) of countering strategy**
**Exposing industry conduct and false claims**	22	[26, 28, 32, 61–63, 65, 67–81]
**Accessing decision-makers**	10	[26, 62, 63, 65, 66, 68–70, 79, 80]
**Generating and using evidence**	10	[28, 32, 65, 66, 68, 74, 79, 81–83]
**Filing a complaint and taking legal action**	8	[27, 63, 64, 66, 76, 81, 84, 85]
**Mobilising coalition and potential supporters**	8	[61–63, 65, 68, 70, 74, 78]
**Additional: Venue-shifting**	1	[61–63, 65, 68, 70, 74, 78, 86]

^a^The total number is higher than the number of included papers because several papers mention more than one counterstrategy

### Counterstrategy 1: Exposing industry conduct and false claims

The most common counterstrategy, found in 22 papers (73%), was to expose industry conduct and disclose false industry claims in order to raise public awareness [32] and pressure decision-makers and government officials [68]. Advocates used a range of tools and platforms, including press conferences and releases, op-eds, media interviews, rallies and advertisements. In Uruguay, advocates published op-eds “denouncing PMI’s attempt to intimidate the government” [69]. In Nigeria, advocates exposed industry attempts to influence government with donations [72], and Nepalese advocates problematised financial links with decision-makers in the media [63]. Mexican advocates criticised tobacco industry’s “intense lobby[ing] of individual legislators, many of whom had previously voted in [its] interests [68], In Costa Rica, a press conference was organised to denounce the Health Minister’s private meeting with industry representatives [65] and in the Philippines, media advocacy and daily rallies were organised to show how a newly introduced bill mirrored industry positions [79].

Exposing industry conduct went beyond industry attempts to directly influence decision-makers and government officials: In US states [70, 73, 74] and in Thailand [26], advocates revealed industry links with third parties and front groups. In Nigeria, health advocates drew attention to the industry’s motives behind CSR activities [72]. In the context of Thai advocates’ mobilisation against an industry conference, “at least 300 articles”, as well as radio and TV stations covered the case [62]. In Mexico City, advocates launched media campaigns during the policy implementation stage in response to British American Tobacco’s attempts to spread confusion over local and federal laws [67]. US advocates showed how industry claims that menthol bans were racist (as they would lead to more contact between Black men and the police) were “disingenuous” [71].

US state-level case studies [73–76] document the use of advertisements at critical moments in the policy process. For example, in Minnesota, advocates countered false industry claims around second-hand smoke by sponsoring an advertising campaign that framed second-hand smoke as a health issue [73]. In Oregon, advocates paid for an ad “listing the people and organisations that supported Measure 44 [a tobacco taxation bill] on one side, and the tobacco industry as the only opposition on the other side” [76].

Exposing industry claims also allowed re-framing of debates where the “tobacco lobby had managed to divert the argument from health concerns to other issues” [77]. For instance, Thai advocates sought to avoid a US-Thai dispute only being narrowly discussed as a trade conflict (instead of a public health issues) due to it being an industry attempt to develop its local cigarette market [61]. In the US, advocates worked to “shift debate about smoking away from the rugged individualism of Marlboro Country onto grounds that legitimised greater government intervention” [81].

### Counterstrategy 2: Accessing decision-makers

Ten studies (33%) reported one or more instances in which advocates approached decision-makers to counteract TII. The purpose of this was to “alert” [63], “inform” [68] or “educate” [26, 63, 70] policymakers. For instance, in Costa Rica, advocates “lobbied legislators to argue the Constitution provides a right to health and not a right to smoke” [65] and provided the Health Ministry with legal advice [66]. Thai advocates attending an official meeting, “presented information to convince [the Ministry of Commerce] and other agencies to continue the ENDS ban” [26], while in Uruguay, advocates met with government representatives “to argue for maintaining the regulations”[69]. In Mexico, advocates “provided evidence to legislators” debunking tobacco companies’ and tobacco growers’ associations claims about job losses and negative effects on farmers [68].

Advocates also sought to put decision-makers under direct or indirect pressure. In Thailand, they “lobbied the [Ministry of Education] intensely until [it] decided to return the donation” [62] from Philip Morris, and in Mauritius, they repeatedly wrote letters to a government official calling out industry sponsorship activities and government involvement [80]. In Nepal, advocates “organized meetings with legislators, media, bureaucrats, and the Kathmandu Metropolitan City Mayor to pressure [the Commerce and Supplies Minister]” [63]. In Costa Rica, advocates “lobbied legislators to pressure [a legislator] to withdraw the substitute language [they had introduced]” [65], while Filipino advocates lobbied for the resignation of a senator who “had filed a bill that resembled Philip Morris’ stance” [79].

### Counterstrategy 3: Generating and using evidence

A third of articles (10/30, 33%) described instances where advocates countered industry interference by generating or using evidence. For example, in California, the American Cancer Society conducted a survey that showed public support for smoke-free bars [74] and in an LMIC, an organisation conducted a study on illicit trade which was described as a “game-changer” during a public hearing [32]. In addition to these activities, advocates garnered support from health experts as in Costa Rica, where advocates invited a representative from Panama’s Health Ministry “to testify that a rise in taxes did not increase contraband in Panama”, also recruiting a prominent lawyer to write a legal opinion [65].

More generally, advocates were reported to use evidence-based arguments to respond to industry claims that threatened tobacco control progress [28, 79, 81, 82]. Advocates provided evidence in response to common industry claims about tobacco taxes in the Philippines, Ukraine and Mexico [68, 79]. For example, Ukrainian advocates shared data showing that cigarette prices in neighbouring countries were higher meaning that a tax increase would decrease cigarette smuggling out of Ukraine [79]. In Costa Rica, advocates shared information on countries that had adopted pictorial health warnings with the Health Ministry [66], and US advocates used “economic data showing that bar and restaurant revenues actually increased following the passing of smoke-free workplace laws” [83].

### Counterstrategy 4: Filing a complaint and taking legal action

Close to one in three articles (9/30, 30%) documented how advocates used complaints and legal tools to counter TII. The targets of such actions varied significantly across jurisdictions. In the US context, advocates “filed a complaint with the Federal Communications Commission (FCC) calling for the Fairness Doctrine to be applied to cigarette advertising” [81]. Another complaint was filed with the FCC against the ten largest radio and television stations in Oregon to enforce compliance with existing regulation [76]. Finish advocates complained to the Chancellor of Justice about TII [84] and in Costa Rica, advocates filed a complaint with the Health Ministry about tobacco companies spreading misinformation [66].

Court cases were also documented: Nepalese advocates repeatedly filed Supreme Court cases regarding violations of executive and supreme court orders on tobacco advertising, promotion and sponsorship (TAPS) [27, 64] and in 2017, they “demand[ed] the rejection of a case filed by [a tobacco company] and full implementation of the 90% [pictorial health warnings]” [63]. In Niger, a tobacco control organisation used its right to sue tobacco companies for violating the advertising ban [85].

### Counterstrategy 5: Mobilising coalition and potential supporters

Over a quarter of articles (8/30, 27%) described how advocates countered industry interference by mobilising and strengthening a pro-tobacco control coalition and teaming up with potential supporters. Thai advocates mobilised against a large industry conference and “youth demonstrated at the event site” [62]. In California, advocates organised community activities to show that industry solicited testimonials “did not reflect the broader public sentiment that favoured smoke-free bars” [74]. Colombian advocates organised workshops with tobacco vendors and gave them stickers “to display for public education about not selling individual cigarettes” [78]. In context of increasing pre-emption bills, US health organisations established a task force which facilitated grassroots mobilisation, and sought partnerships with the legal community [70].

To build credibility and strengthen their case, advocates also drew on leading public figures [61], prominent national and international lawyers [65], and senior religious leaders [61]. They also closely worked with relevant government stakeholders [61, 62, 65, 70],

Advocates also sought and received support, including financial and technical support, from international public health organisations and activists [61–63, 65, 68, 78]. For example, a Costa Rican civil society organisation “worked closely with an international coalition of health groups, led by the US-based Campaign for Tobacco-Free Kids… and collaborated with the Pan American Health Organisation” [65] and in Colombia, advocates received, for example, support from the Bloomberg Initiative, helping them to push for policy implementation [78].

### Additional counterstrategy: Venue-shifting

One paper [86] described that when US tobacco control advocates were unsuccessful in pushing state laws due to industry influence, they turned to the local level, seeking to pursue local ordinances. By this they used their “organisational strengths […] as well as the weaknesses of the tobacco industry” [86].

## What action-based industry strategies do advocates counter with what counterstrategies?

Advocates repeatedly countered five action-based industry strategies (see Table 2). Illicit trade is the only strategy included in the PDM that did not feature in the data. We did not identify additional industry strategies not captured by the PDM.

**Table 2:** Action-based industry strategies

**PDM action-based strategies **[22, 25]	**No of papers**^**a**^	**Papers mentioning example(s) of countering action-based strategy**
**Direct Access and Influence**	17	[26, 28, 32, 63, 65, 66, 68–70, 72–74, 78, 79, 81, 82, 86]
**Information Management**	15	[32, 63, 65–68, 71, 73–79, 82]
**Coalition Management**	12	[26, 28, 63, 65, 66, 68, 70, 73, 74, 78, 81, 83]
**Reputation management**	9	[27, 62, 64, 72, 74, 78, 80, 84, 85]
**Litigation**	4	[61, 63, 70, 74]
**Illicit trade**	0	n/a

^a^The total number is higher than the number of included papers because several papers mention more than one industry strategy that was countered

When mapping the industry strategies against the advocates’ counterstrategies, we found that advocates responded to almost all industry strategies with almost all counterstrategies (Table 3, see Additional file 2 for the references per cell). While for some industry strategies, several counterstrategies were prominent, in others, specific counterstrategies were dominant. At times, counterstrategies were – as industry strategies – also used in parallel.

**Table 3:**
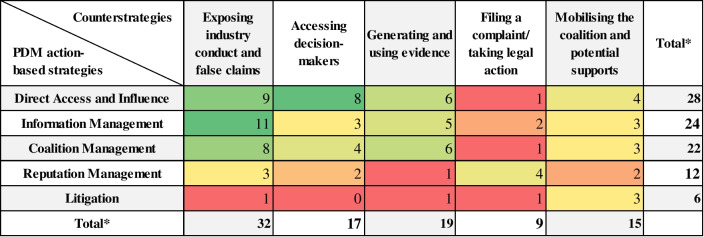
PDM action-based strategies and counterstrategies and number of included studies

^a^The total number is higher than the number of included papers because several papers mention more than one instance of countering, counterstrategy and/or industry strategy that was countered

In the following, we summarise the industry strategies and how these were countered.

### Direct access and influence

In over half of the papers (17/30, 57%) advocates countered industry attempts to directly influence policymaking. Countering activities to lobbying of and links with key stakeholders in governments and legislatures were frequently reported [28, 63, 66, 70, 74, 78, 79, 81, 86]. Advocates also responded to attempts of the industry and its allies to participate in consultations [32, 82] or meetings [26, 73], and their use of threats [68-70] and incentives [63, 72] to influence policymakers and -making.

These industry tactics were most frequently countered by exposing industry conduct and claims (Counterstrategy 1): Among others, in Costa Rica, advocates exposed a minister’s meeting with the industry [65] while financial links with decision-makers were made public in Nepal [63]. In Uruguay and Mexico, advocates exposed industry attempts to intimidate the government [68, 69] and Nigerian advocates revealed industry attempts to influence policymaking through donations [72].

The second most frequently mentioned approach to countering Direct Access and Influence was ‘Accessing decision-makers’ (Counterstrategy 2) which meant using a strategy similar to the one countered: it involved “lobbying” key policymakers [63, 65, 66, 70, 78, 79], meeting them [63, 66, 69] or writing them letters [69]. This strategy was at times used in parallel with Counterstrategy 1 [63, 65, 78].

Some studies reported how advocates worked with evidence (Counterstrategy 3) and mobilised supporters (Counterstrategy 5) to counter industry attempts to directly influence policymaking. For example, when industry threatened to cease operations in the country, advocates provided evidence, “showing that the tobacco industry´s investment in Mexico was insignificant” [68] (Counterstrategy 3). Where the industry shared exaggerated figures linked to illicit trade or taxes with policymakers, advocates countered by sharing results from their own study [32] and hiring international experts to provide evidence [65] (Counterstrategy 3). In both cases, evidence was provided to policymakers, meaning that Counterstrategies 2 and 3 were combined. The latter example can also be seen as a way of expanding the tobacco control network (Counterstrategy 5). In another instance, when industry met with decision-makers, advocates responded by working together and seeking support from international groups (Counterstrategy 5), while also exposing industry conduct (Counterstrategy 1) and accessing decision-makers (Counterstrategy 2) [65].

Only one study reported a complaint filed (Counterstrategy 4) in response to excessive industry influence on US-policymaking: the complaint with the FCC explored “alternative means” to strengthen tobacco control [81].

### Information management

Fifteen articles (50%) included examples of advocates countering the industry’s management of information to influence policymaking, including attempts to divert debates away from health to other issues [77, 82], generate and disseminate industry-friendly information [63, 65, 73] and spread misleading or false claims [32, 63, 66–68, 71, 75, 78, 79]. In a few instances, advocates also countered front groups disseminating industry-favourable information [65, 74].

By far the most prominent strategy advocates used to counter information management was ‘Exposing industry conduct and false claims’ (Counterstrategy 1). This included media campaign to counter industry’s misleading claims on second-hand smoke [73] and new policies [67]. When industry advertisements in Oregon asserted that “healthcare provider… would use the cigarette tax money to line their pocket”, advocates highlighted in a print advertisement that the initiative was supported by popular voluntary health agencies [76]. When industry launched statements claiming that a tax raise would increase illicit trade using Canada as “inaccurate example”, advocates shared letters with media outlets [68]. The letters were also shared with policymakers (Counterstrategy 2) and one was from international public health organisations (Counterstrategy 5), showing how several strategies were combined.

Counterstrategy 3 (Generating and using evidence) was reported in five studies. For example, in Costa Rica, the industry paid legal experts to write favourable opinions which advocates countered by hiring a constitutional lawyer to debunk these claims [65]. In the Philippines and the Ukraine, advocates countered industry claims that tax increases would, among others, increase tobacco smuggling and illicit trade with evidence showing that this was extremely unlikely [79]. Similarly, in Mexico when the tobacco growers’ association stated that a tax increase would affect them negatively, advocates provided evidence showing that the “economic costs of treating tobacco-related health issues are exponentially higher than the economic detriment to tobacco producers resulting from reduced consumption” [68].

The other counterstrategies were reported in two to three studies. For example, in response to false industry claims, advocates “met and alerted” policymakers (Counterstrategies 2) [63]. When the industry made false claims during policy formulation and implementation, advocates worked with international health groups and relevant government stakeholders (Counterstrategy 5) [68, 78]. Lastly, when the industry spread misleading information among merchants about the new policy, Costa Rican advocates complained to a Ministry [66] (Counterstrategy 4).

### Coalition management

Twelve papers (40%) documented instances where advocates worked against industry coalition management before and after policy adoption. Advocates responded to industry efforts to work with industry bodies and associations [63, 73, 78], a local tobacco monopoly [28], and through hospitality groups [28, 63, 65, 66, 74, 83]. They also, for example, countered activities of a tobacco industry employee union [63], an industry-aligned tobacco growers’ association [68] and a “nominally independent” organisation promoting e-cigarettes [26].

The most prominent counterstrategy to coalition management was to ‘Exposing industry conduct and false claims’ (Counterstrategy 1). For example, in US states [70, 73, 74] and in Thailand [26], advocates revealed that third parties were linked to the industry (although in most cases, the nature of those links was not specified).

‘Generating and using evidence’ (Counterstrategy 3) was the second most prominent response to coalition management. In the US, “widely accepted anecdotal claims” pushed by an industry front group, were countered with a survey, conducted by advocates [74]. Mexican advocates countered claims made by the tobacco growers’ association on the likely effect of tax increases with evidence which they shared with the media (Counterstrategy 1) and policymakers (Counterstrategy 2) [68].

Counterstrategies 2 and 5 were also used repeatedly and could appear together, i.e., educating policymakers about the false claims made by front groups (Counterstrategy 2), also receiving support from international public health groups (Counterstrategy 5) [63, 65]. In one study, advocates complained to a Ministry (Counterstrategy 4) regarding a pamphlet which had been written by the industry and endorsed by a “long-time industry ally and hospitality front group” [66].

### Reputation management

Thirty percent of the papers (9/30) described instances where advocates countered tobacco companies’ attempts to present themselves in a favourable light, including attempts to appear as if they were a government partner [62, 72]. Most often, countered industry efforts sought to undermine or circumvent policy implementation [27, 64, 74, 78, 84, 85].

The most frequent response to reputation management linked to complaints and legal actions (Counterstrategy 4) [27, 64, 84, 85]. For instance, in Finland, when the industry sought to sponsor a yachtsman during an around the world race, circumventing the existing regulations, advocates complained to the Chancellor of Justice [84]. In Nepal, in response to a 5-year deal between industry and the national cricket association [27] as well as a televised concert and free distribution of cigarettes to youth [64], advocates filed cases at the Supreme Court asking the government to enforce existing TAPS regulations.

Counterstrategy 1 (Exposing industry conduct and false claims) were mentioned in three studies as responses to companies’ attempts of managing its reputation. For example, in Nigeria, advocates tried to “expose business motives behind [the] CSR [programmes]” [72] and in Mauritius, advocates sought to expose the industry’s undergraduate scholarship schemes, while also “writ[ing] to the government representative that was on stage with the tobacco industry to shame them” [80] (Counterstrategy 2). Counterstrategy 2 was also employed by Thai advocates when they lobbied a Ministry that had accepted an industry donation. To counter an industry public relations campaign, US advocates worked together and engaged, among others, in community activities (Counterstrategy 5), while also running a survey to debunk industry claims [74] (Counterstrategy 3).

### Litigation

Only four studies (13%) reported advocates’ responses to industry’s legal action: two linked to litigation at the subnational level in the US [70, 74], one to the Supreme Court in Nepal [63] and one to an investigation into unfair trading practices [61].

In three studies, advocates mobilised a coalition of supporters (Counterstrategy 5) in light of industry’s legal actions. For example, in response to the industry’s legal challenges to state pre-emption, advocates established links with the legal community [70] and in the case of the investigation into unfair trading practices, Thai advocates received support from international advocates and organisations, relevant public personalities as well as religious leaders [61].

All other counterstrategies were identified in one study each, except ‘Accessing decision-makers’ (Counterstrategy 2) which was not used to counter tobacco industry litigation. In the trade investigation, advocates also “sought maximum media publicity for their cause” [61] (Counterstrategy 1). To counter industry repeal attempts (as well as the public relations campaign – see above), US advocates generated evidence in the form of a survey [74] (Counterstrategy 3) and while a law suit was pending, Thai advocates “lobbied” political leaders and judges [63] (Counterstrategy 2).

## Effectiveness of countering activities

Having identified how advocates countered which industry strategy, we next assessed if the counterstrategies were described as effective, either with immediate, short-term effects (outcomes); and/or long-term effects (impact).

Several studies described outcomes (short-term effects) of countering activities and we found some differences between counterstrategies: four papers [76, 78, 81, 84] reported instances where complaints or legal action (Counterstrategy 4) resulted in a favourable outcome: The FCC agreed with advocates’ complaints [76, 81], a ministry revoked an authorisation for which advocates had petitioned [78] and a conditional fine was imposed in response to advocates’ complaints [84]. In other studies, there was no immediate effect of such actions, and court responses were described as slow [63] – in one instance, it took around 3 years [64] – or a ministry did not respond [66].

Some studies reported positive outcomes where advocates exposed industry conduct and claims (Counterstrategies 1) and accessed decision-makers (Counterstrategy 2). For example, in Thailand, both counterstrategies used in parallel, resulted in a ministry returning a donation from the industry and advocates’ protest against an industry conference reduced the number of attendees [62]. Also, in the Philippines both strategies were used together, leading the President to push for a senator’s resignation [79]. Elsewhere, a media campaign led a minister to write a letter opposing the exposed industry activities [27]. In another instance, campaigning by advocates resulted in “strongly worded veto messages” [70]. In other studies, the outcomes of media campaigns [65, 73] or lobbying policymakers [65, 66, 80] were not made explicit.

Other counterstrategies were also described as effective: For example, the use of evidence (Counterstrategy 3) on illicit trade “changed the mentality of parliamentarians” [32]. Elsewhere, advocates evidence-based arguments contributed to “legislators realiz[ing] that the industry was trying to intimidate them and [becoming] more hostile to industry interests” [68]. The effect of mobilising a coalition (Counterstrategy 5) was only described together with other counterstrategies. For example, when letters, including one from international public health organisations were disseminated to media outlets, leading to newspapers articles (Counterstrategy 1) which reached the government ministries, the “target audience” [68].

Looking at the impact (i.e., the longer-term effects) of countering strategies, only a few studies outlined direct links between advocates’ countering actions and policy change: In one instance, a complaint led to a tightening of an advertising ban some years later [84]. Most studies stated more generally that advocates’ countering work was “successful” [26, 27, 62, 68, 77, 83, 86] or “effective” [26, 28, 62, 63, 77, 78, 83] in contributing to tobacco control policymaking. However, overcoming TII in policymaking also required committed decision-makers and public officials [28, 62, 63, 65–69, 71, 74, 79–81] as well as close collaborations and coordination between government and international and local public health groups [61, 63, 65, 67, 72]. Two studies reported that a multi-sectoral approach could also facilitate working against industry strategies [27, 69]. In LMICs, international technical support, training and funding were cited as key for countering TII [27, 62–69, 72, 78].

## Discussion

Compared to the rich literature into TII [22–25], the body of peer-reviewed literature on advocates countering TII is very small. However, this scoping review indicates that such literature appears to be growing, with half of the included studies being published post-2015. There was also a notable shift from HIC- to LMIC-focused publications over time which could reflect the tobacco industry’s increasing focus on LMICs [87] as well as growing academic interest in LMIC-focused work.

This review found that civil society actors use a range of strategies in response to tobacco industry attempts to influence policymaking. It identifies five counterstrategies to TII: 1. Exposing industry conduct and false claims, 2. Accessing decision-makers, 3. Generating and using evidence, 4. Filing a complaint and taking legal action, and 5. Mobilising coalition and potential supporters. While these strategies were developed inductively, they partially overlap with and expand the four activities found in a previous interview-based study with advocates’ from eight LMICs (generating and compiling data and evidence; accessing policymakers and restricting tobacco industry access; working with media; engaging in a national tobacco control coalition) [32]. Coalition building and mobilising potential supporters was the least commonly reported strategy used in direct response to TII, however, it was identified as a key facilitator for effective countering work more generally [27, 62–69, 72, 78].

We also identified an additional counterstrategy: venue-shifting which not only – as in the example included in this review [86] – refers to shifting between levels of government but can also involve shifting within institutions or between policy terrains [88]. In the tobacco control literature, venue-shifting has often been associated with industry efforts [89–91], but studies in other areas, including women’s rights [92], HIV [93] and forestry [93, 94], illustrate venue-shifting as an advocacy strategy.

This review indicates that advocates counter a wide range of tobacco industry tactics. The studies described instances of countering five of the six action-based strategies identified in the PDM [22, 25] (Coalition Management, Information Management, Direct Access and Influence, Litigation and Reputation Management). Illicit trade was the only PDM strategy not found in the dataset. This could be because, prior to the Protocol to Eliminate Illicit Trade in Tobacco Products which entered into force in 2018, other areas of the WHO FCTC received more attention [95]. For instance, the MPOWER measures do not include illicit trade [96]. Furthermore, given that illicit trade is an illegal activity, it is difficult to research, and the tobacco industry also goes to great length to conceal its involvement which is now well-documented [97–99]. It could therefore be hard for advocates to recognise such activities which could impede countering action.

To date, the literature has emphasised the industry’s ability to tailor its strategies to a context and exploit local specificities [100–105]. This review suggests that advocates also employ their tactics flexibly, using almost all counterstrategies to counter almost all TII strategies. Furthermore, all counterstrategies are similar to one or two industry strategies (Mobilising coalition and potential supporters and Coalition Management; Exposing industry conduct/ Generating and using evidence and Information Management; Accessing decision-makers and Direct Access and Influence; Filing a complaint and taking legal action and Litigation) which could reflect advocates’ intensions to beat the industry at its own game [106]. Only Reputation Management has no counterstrategy equivalent, however, future work could explore whether and how advocates work on their image, for example, when attacked by the industry [107].

We also analysed how the peer-reviewed studies described the effectiveness of the advocates’ countering attempts. Looking at the outcome, some studies describe a positive effect of countering. Far fewer studies report a negative outcome and often, no clear short-term effect of countering was mentioned. This could reflect that most studies focused on the tobacco control journey of a country or state meaning that advocates’ activities and their consequences were only described in more detail where needed for the overall story. Furthermore, many studies (e.g., [26–28, 63, 65, 69, 72–74, 76, 79]) covered cases of “success”, for example, a policy was adopted despite TII. These might be more likely to get researched and published than those of no or limited “success” which could be a facet of publication bias [108]. In scenarios with overall progress, countering could be more effective than elsewhere.

The review has several limitations. First, it is limited to peer-reviewed articles. While this served to ensure a high standard of evidence, advocates’ work is rarely written-up in peer-reviewed form. We did not include information found, for example, in media reports, in organisational documents, conference presentations, commentaries and interviews. In a next step, one could repeat the searches covering grey literature using the identified counterstrategies as a starting point.

Second, this study was limited to peer-reviewed papers written in English, German, and Spanish as members of the research team were proficient in these languages. However, only a very small number of search results were excluded due to the language criterion. Furthermore, even with broad and additional searches, we might have missed some relevant studies.

Another limitation is that there was often little detail about countering activities and their effectiveness. In most cases, more attention is paid to TII and overall tobacco control progress. To get more in-depth insights, future work could engage with advocates’ perspectives on “success” and “failure”. We also did not explore common counterarguments and their effectiveness which could be explored in future work.

This review also only captures reactive work of civil society actors that are explicitly described as such and not, for example, proactive strategies and where it was not stated if a strategy was re- or proactive. We also only focus on countering in the context of policymaking. Future work could look at tobacco control advocacy more holistically, exploring, among other, how advocates decide on and adapt their approach over the course of public health policymaking and beyond. Further research could also look at countering TII more broadly, not only looking at civil society action but also those of decision-makers and public officials.

We encourage researchers and research funders to continue moving beyond studying TII and direct more attention to studying the role of tobacco control advocacy in countering TII and policymaking more generally. Exploring more cases with limited or no progress would be particularly valuable given the current focus on “success” stories. Such work would allow learning from the breadth and depth of experiences of civil society members and organisations in tobacco control and could also contribute to work beyond tobacco control [109].

The categorisation of counterstrategies proposed here offers a starting point to discuss advocates’ activities of countering TII. It could be developed further into an evidence-based tool for capacity-building purposes which complement existing material on TII. This could stimulate exchange about experiences of addressing TII which advocates identified as needed for capacity building [32].

## Conclusions

TII remains a key obstacle to tobacco control progress. While a large number of studies have led to a detailed understanding of tobacco industry strategies to prevent, weaken, and undermine policies, we know far less about the role of civil society in countering TII. This scoping review shows that the peer-reviewed literature describing specific instances of advocates countering TII is scarce and documented examples often lack detailed descriptions. Our analysis suggests that civil society actors employ several strategies to counter TII in its different forms and use their tactics flexibly. More research is needed to better understand the effects of advocates’ actions, also considering cases with limited or no tobacco control progress. This could stimulate discussions about and facilitate learning from past experiences.

## Supplementary Information

**Additional file 1.** List of articles included in the scoping review.

**Additional file 2.** PDM action-based strategies and counterstrategies - References per cell for Table 3.

**Additional file 3.** Preferred Reporting Items for Systematic reviews and Meta-Analyses extension for Scoping ReviewsChecklist.

## Data Availability

N/A.

## Abbreviations

FCC: Federal Communications Commission
FCTC: Framework Convention on Tobacco Control
HICs: High-income countries
LMICs: Low- and middle-income countries
PDM: Policy Dystopia Model
TAPS: Tobacco advertising, promotion and sponsorship
TII: Tobacco industry interference
WHO: World Health Organization
