# Supplementary material for: Advocacy counterstrategies to tobacco industry interference in policymaking: a scoping review of peer-reviewed literature
Source: Global Health. 2023 Jun 21;19:42. doi: 10.1186/s12992-023-00936-7 (PMC10286487; doi:10.1186/s12992-023-00936-7)
Supplement: Supplementary file 1 — Additional file 1. List of articles included in the scoping review. [file 12992_2023_936_MOESM1_ESM.pdf]

Additional File 1: Articles included in the scoping review (in chronological order)

Author(s)	Year	Title	Data sources (according to the authors or, if not available, our reading)	Geographical focus of paper (and of example(s) if different) and income group
**Macdonald HR/ Glantz SA**	1997	Political Realities of State-wide Smoking Legislation: The Passage of California's Assembly Bill 13	Interviews, data from legislative hearings and debates, public documents, letters and personal communication, internal memoranda, newspaper articles.	California, USA High-income country (HIC)
**Goldman LK/ Glantz SA**	1999	The passage and initial implementation of Oregon's Measure 44	Interviews, public records, internal memoranda, newspaper articles	Oregon, USA HIC
**Sato H**	1999	Policy and politics of smoking control in Japan	Government documents, Japan Monopoly Public Corporation documents	Japan (Canada) HIC
**Givel MS/ Glantz SA**	2000	Tobacco Control and Direct Democracy in Dade County, Florida: Future Implications for Health Advocates	Internal industry documents, public records, newspaper articles, documents from health organisations, interviews	Florida, USA HIC
**Bero L et al.**	2001	Science in Regulatory Policy Making: Case Studies in the Development of Workplace Smoking Restrictions	Written public commentaries and hearing transcripts, including substantive letters, reports and the first copies of duplicate letters	Maryland and Washington, USA HIC
**Chantornvong S/ McCargo D**	2001	Political Economy of Tobacco Control in Thailand	Interviews, reports, newspaper articles	Thailand Lower-middle income country (LMIC)
**Magzamen S/ Glantz SA**	2001	The new battleground: California's experience with smoke-free bars	Interviews, newspaper articles, internal industry memoranda, personal correspondence, public documents, legislative meetings	California, USA HIC
**Hiilamo H**	2003	Tobacco industry strategy to undermine tobacco control in Finland	Internal industry documents	Finland HIC
**Tsoukalas T/ Glantz SA**	2003	The Duluth Clean Indoor Air Ordinance: Problems and Success in Fighting the Tobacco Industry at the Local Level in the 21st Century	Interviews, newspaper articles, internal industry documents, reports (government and third sector), academic literature	Duluth, Minnesota (USA) HIC
**Bailey C**	2004	From "Informed Choice" to "Social Hygiene": Government Control of Cigarette Smoking in the US	Policy documents, including government reports and speeches, newspaper articles, other grey literature, academic literature	USA HIC
**O'Dougherty M et al.**	2010	Communicating With Local Elected Officials: Lessons Learned From Clean Indoor Air Ordinance Campaigns	Interviews	USA HIC
**Crosbie E et al.**	2011	Strong advocacy led to successful implementation of smokefree Mexico City	Policy documents, newspaper articles. Interviews	Mexico City Upper-middle income country (UMIC)
**Tumwine J**	2011	Implementation of the Framework Convention on Tobacco Control in Africa: Current status of legislation	WHO data. legal documents, supplementary: academic articles, internal industry documents, newspaper articles	Several African countries (Niger) Low-income country (LIC) (Niger)
**Charoenca N et al.**	2012	Success counteracting tobacco company interference in Thailand: An example of FCTC implementation for low- and middle-income countries	Documents (including historical records advocates collected, internal industry documents and annual	Thailand UMIC

			reports), company websites, interviews	
**Lane C/ Carter M**	2012	The role of evidence-based media advocacy in the promotion of tobacco control policies	Reports, internal records	Mexico UMIC
**Crosbie E et al.**	2016	Costa Rica's implementation of the Framework Convention on Tobacco Control: Overcoming decades of industry dominance	Policy documents, newspaper articles, interviews	Costa Rica UMIC
**Crosbie E et al.**	2017	The importance of continued engagement during the implementation phase of tobacco control policies in a middle-income country: the case of Costa Rica	Policy documents, newspaper articles, interviews	Costa Rica UMIC
**Crosbie E et al.**	2018	Defending strong tobacco packaging and labelling regulations in Uruguay: transnational tobacco control network versus Philip Morris International	Policy documents, newspaper articles, interviews	Uruguay HIC
**Nakkash RT et al.**	2018	The passage of tobacco control law 174 in Lebanon: reflections on the problem, policies and politics	Historical records of government decisions, documentation of advocacy campaign, interviews	Lebanon UMIC
**Uang R et al.**	2018	Tobacco control law implementation in a middle-income country: Transnational tobacco control network overcoming tobacco industry opposition in Colombia	Newspaper articles, policy and legal documents, NGO reports, interviews	Colombia UMIC
**Egbe CO et al.**	2019	Role of stakeholders in Nigeria's tobacco control journey after the FCTC: lessons for tobacco control advocacy in low-income and middle-income countries	Interviews, newspaper articles, copies of public hearing proceedings	Nigeria LMIC
**Bhatta DN et al.**	2020 a	Exceeding WHO Framework Convention on Tobacco Control (FCTC) Obligations: Nepal Overcoming Tobacco Industry Interference to Enact a Comprehensive Tobacco Control Policy	Newspaper articles, policy documents, interviews	Nepal LMIC
**Bhatta DN et al.**	2020 b	Defending comprehensive tobacco control policy implementation in Nepal from tobacco industry interference (2011–2018)	Newspaper articles, policy documents, interviews	Nepal LMIC
**Bhatta DN et al.**	2020 c	Tobacco control in Nepal during a time of government turmoil (1960–2006)	Tobacco industry documents, newspaper articles, interviews	Nepal LMIC
**Crosbie E/ Schmidt LA**	2020	Preemption in Tobacco Control: A Framework for Other Areas of Public Health	State reports, academic literature	USA HIC
**Matthes BK et al.**	2020	Needs of LMIC-based tobacco control advocates to counter tobacco industry policy interference: Insights from semi-structured interviews	Interviews	Eight LMICs (not specified)
**Patanavanich R/ Glantz SA**	2020	Successful countering of tobacco industry efforts to overturn Thailand's ENDS ban	Newspaper articles, reports, information from websites and social media platforms of relevant groups, meeting minutes, letters submitted to government agencies	Thailand UMIC
**Bosma LM et a.**	2021	Restricting Sales of Menthol Tobacco Products: Lessons Learned from Policy Passage and Implementation in Minneapolis, St. Paul, and Duluth, Minnesota	Interviews	Minneapolis, St. Paul, Duluth, Minnesota (USA) HIC
**Hoe C et al.**	2021	The battle to increase tobacco taxes: Lessons from Philippines and Ukraine	Interview, documents	Philippines Ukraine LMIC (both)
**Kusi-Ampofo, O**	2021	Negotiating Change: Ideas, Institutions, and Political Actors in Tobacco Control Policy Making in Mauritius	Government documents, media reports, archival studies, grey literature, published books and articles, interviews	Mauritius UMIC

Income group: based on World Bank data and year of publication (accessed on 16/03/2022 through https://datatopics.worldbank.org/world-development-indicators/the-world-by-income-and-region.html)
