# Supplementary material for: Advocacy counterstrategies to tobacco industry interference in policymaking: a scoping review of peer-reviewed literature
Source: Global Health. 2023 Jun 21;19:42. doi: 10.1186/s12992-023-00936-7 (PMC10286487; doi:10.1186/s12992-023-00936-7)
Supplement: Supplementary file 2 — Additional file 2. PDM action-based strategies and counterstrategies - References per cell for Table 3. [file 12992_2023_936_MOESM2_ESM.pdf]

Additional File 2: PDM action-based strategies and counterstrategies - References per cell for Table 3

PDM instrumental strategies / counter strategies	Exposing industry conduct and false claims	Accessing decisionmakers	Generating and using evidence	Filing a complaint/ taking legal action	Mobilising coalition and potential supporters
**Direct Access and Influence**	Bhatta et al. 2020a, Crosbie et al. 2016, Crosbie et al. 2018, Egbe et al. 2019, Hoe et al. 2021, Lane/ Carter 2012, Magzamen/ Glantz 2001, Nakkash et al. 2018, Tsoukalas/ Glantz 2003	Bhatta et al. 2020a, Crosbie/ Schmidt 2020, Crosbie et al. 2016, Crosbie et al. 2017, Crosbie et al. 2018, Hoe et al. 2021, Lane/ Carter 2012, Patanavanich/ Glantz 2020	Bero et al. 2001, Crosbie et al. 2016, Crosbie et al. 2017, Lane/ Carter 2012, Matthes et al. 2020, Nakkash et al. 2018,	Bailey 2004	Bhatta et al. 2020a, Crosbie/ Schmidt 2020, Crosbie et al. 2016, Uang et al. 2018
**Information Management**	Bhatta et al. 2020a, Bosma et al. 2021, Crosbie et al. 2011, Givel/ Glantz 2000, Goldman/ Glantz 1999, Lane/ Carter 2012, Magzamen/ Glantz 2001, Matthes et al. 2020, Sato 1999, Tsoukalas/ Glantz 2003, Uang et al. 2018	Bhatta et al. 2020a, Crosbie et al. 2016, Lane/ Carter 2012,	Bero et al. 2001, Crosbie et al. 2016, Hoe et al. 2021, Lane/ Carter 2012, Magzamen/ Glantz 2001,	Crosbie et al. 2017, Goldman/ Glantz 1999	Crosbie et al. 2016, Lane/ Carter 2012, Uang et al. 2018
**Coalition Management**	Bhatta et al. 2020a, Crosbie/ Schmidt 2020, Magzamen/ Glantz 2001, Lane/ Carter 2012, Nakkash et al. 2018, Patanavanich/ Glantz 2020, Tsoukalas/ Glantz 2003, Uang et al. 2018	Bhatta et al. 2020a, Crosbie et al. 2016, Lane/ Carter 2012, Patanavanich/ Glantz 2020,	Bailey 2004, Crosbie et al. 2016, Magzamen/ Glantz 2001, Lane/ Carter 2012, Nakkash et al. 2018, O'Dougherty et al. 2010,	Crosbie et al. 2017	Bhatta et al. 2020a, Crosbie et al. 2016, Uang et al. 2018
**Reputation management**	Charoenca et al. 2012, Egbe et al. 2019, Kusi-Ampofo 2021	Charoenca et al. 2012, Kusi-Ampofo 2021	Magzamen/ Glantz 2001	Bhatta et al. 2020b, Bhatta et al. 2020c, Hiilamo 2003, Tumwine 2011	Charoenca et al. 2012, Magzamen/ Glantz 2001
**Litigation**	Chantornvong/ McCargo 2003		Magzamen/ Glantz 2001	Bhatta et al. 2020a	Chantornvong/ McCargo 2003, Crosbie/ Schmidt 2020, Magzamen/ Glantz 2001
**Illicit trade**					
